# Supplementary material for: De novo main-chain modeling for EM maps using MAINMAST
Source: Nat Commun. 2018 Apr 24;9:1618. doi: 10.1038/s41467-018-04053-7 (PMC5915429; doi:10.1038/s41467-018-04053-7)
Supplement: Supplementary file 1 — Supplementary Information [file 41467_2018_4053_MOESM1_ESM.pdf]

Supplementary Infomration

De novo main-chain modeling for EM maps using MAINMAST

Terashi et al.

Supplementary Table 1. Results of MAINMAST on the dataset of 40 simulated maps.

PDBID	Top-scoring CαRMSD(Å)[a]	Best CαRMSD(Å)[b]	CLICK-RMSD(Å)[c]	CLICK-SO (%)[d]	CLICK-TS[e]
1a1x	0.95	0.95	1.73	93.40	1.00
1auu	1.43	1.24	2.18	72.73	1.00
1b25	1.68	1.36	1.87	93.78	1.00
1ba3	1.75	1.06	2.00	86.79	1.00
1ejd	2.80	2.02	2.74	68.12	0.91
1ewf	2.38	1.70	2.53	78.89	0.76
1h12	2.43	2.06	2.87	60.40	1.00
1h70	2.39	1.77	2.80	67.06	0.95
1h8p	1.62	1.23	2.30	86.36	1.00
1i5p	1.69	1.62	1.78	96.46	1.00
1igd	1.58	1.12	1.68	96.72	1.00
1iwm	1.80	1.48	2.10	92.66	1.00
1j0p	1.13	1.13	2.06	80.56	1.00
1lkt	1.88	1.19	2.16	89.42	1.00
1m3y	1.71	1.39	1.54	97.34	1.00
1mkn	1.31	1.12	1.72	88.14	1.00
1n7v	1.65	1.52	1.87	93.78	1.00
1oai	2.34	1.51	1.89	94.35	1.00
1ogq	1.88	1.42	2.75	60.70	1.00
1p9h	1.21	1.09	1.73	94.57	1.00
1plq	1.86	1.48	2.34	82.58	1.00
1ppr	2.07	1.91	2.18	86.16	1.00
1qsa	2.52	2.22	2.98	31.96	0.17
1rg8	1.60	1.09	1.67	95.74	1.00
1tl2	1.40	1.29	1.60	96.60	1.00
1v3w	1.18	1.14	2.28	92.49	1.00
1vbw	1.90	1.21	2.10	94.12	1.00
1vq8	1.63	1.48	2.26	93.04	1.00

1w6s	2.02	1.52	2.90	59.16	0.94
1wru	1.70	1.32	2.35	61.36	0.92
1yfq	1.77	1.39	2.73	61.99	0.82
2bf6	2.10	1.78	2.80	63.35	0.94
2bmo	1.07	1.02	1.70	94.57	1.00
2dpf	1.94	1.21	1.76	96.40	1.00
2eiy	2.11	1.32	2.39	77.78	1.00
2erl	1.57	1.30	2.57	67.50	1.00
2hba	1.77	1.42	1.95	61.54	1.00
2hnu	2.92	1.59	2.15	82.72	1.00
3c7x	1.43	1.27	2.25	88.27	1.00
3hms	1.33	0.98	1.87	95.60	1.00
**Average**	1.79	1.40	2.18	81.88	0.96

a, the root mean square deviation (RMSD) of Cα atoms between the first (top-scoring) MAINMAST model and the native structure; **b**, the lowest CαRMSD among 2688 MAINMAST models generated for the map; **c**, the RMSD of aligned region of the first MAINMAST model computed by the CLICK server; **d**, the percentage of the Cα atoms in the first MAINMAST model that are within 3.5 Å of the aligned Cα atoms in the target model computed by the CLICK server; **e**, the topological similarity score between the first MAINMAST model and the native structure computed by CLICK. The topology score considers the direction on the sequence of the matched fragments of two structures compared. It ranges from 0 to 1 with 1 being the maximum score.

Supplementary Table 2. The dataset of 30 experimental maps.

EMDB ID	PDB ID	Name	Resolution (Å)	Length	Contour Level
1461	1qhd-A	Rotavirus VP6 protein	3.8	397	1.04
2513A	4ci0-A	F420-reducing hydrogenase, subunit alpha	3.36	385	0.09
2513B	4ci0-B	F420-reducing hydrogenase, subunit gamma	3.36	228	0.09
2513C	4ci0-C	F420-reducing hydrogenase, subunit beta	3.36	280	0.09
3231	5fmg-K	proteasome 20S core	3.6	194	0.76
3246A	5foj-A	Nanobody	2.8	130	2.67
3246B	5foj-B	Grapevine fanleaf virus	2.8	504	2.67
5185	3j06-A	Tobacco Mosaic Virus	3.3	155	6.42
5495	3j26-A	Sputnik virophage	3.5	508	2
5584	3j31-A	Sulfolobus turreted icosahedral virus	3.9	344	0.05
5764	3j4u-A	Bordetella phage BPP-1	3.5	327	0.025
5778	3j5p-A	TRPV1	3.275	311	7
5925	3j6j-A	Mitochondria Anti-viral Signaling protein, CARD domain	3.64	97	0.3
6219	1pma-A	20S proteasome	4.8	221	4.5
6272	3j9s-A	Bovine rotavirus VP6	2.6	397	0.02
6374	3jb0-D	Bombyx mori cypovirus 1	2.9	292	4
6478	3jbs-A	60S ribosomal protein L6E	2.9	175	0.01
6551	3jcf-A	CorA	3.8	349	0.04
6555	3jci-A	Porcine circovirus 2	2.9	190	10
8011	5gam-D	Small nuclear ribonucleoprotein Sm D3	3.7	82	0.012
8015	5gaq-A	Membrane-inserted form of the nonameric pore forming protein Lysenin	3.1	288	0.07
8116	5ire-A	Zika virus	3.8	501	3.5
2364	4btg-A	Pseudomonas phage phi6	4.3	761	2
2850	5aey-A	ParM	4.3	318	1.8
2867	4uft-B	Nucleoprotein	4.3	377	1
3063	5a6f-C	Slo2.2	4.2	540	0.015
3073	5a79-A	Barley stripe mosaic virus	4.1	177	17
3074	5a7a-A	Barley stripe mosaic virus	4.1	175	20
5155	3iyj-A	Bovine papillomavirus type 1	4.2	481	2.6

5376	3j17-D	Bombyx mori cypovirus 1	4.1	291	2.6

Contour level of a map indicates a density contour for capturing the shape of the target protein that is recommended by the authors of the map.

Supplementary Table 3. Results of MAINMAST on the dataset of 30 experimental maps.

EMDB ID	Resolu-tion (Å)	MAINMAST [a)]					Rosetta (0.8) [b)]		
		Main-chain		Refined					
		Top1	Top10	Top1	Top 10	Coverage (Refined Top 1)	Top 1	Top10	Coverage (Top 1)
1461	3.8	30.5	30.2	30.6	30.6	0.86	17.9	16.8	0.78
2513A	3.36	4.3	4.3	3.8	3.5	0.96	9.9	9.9	0.90
2513B	3.36	9.7	5.1	4.3	2.7	0.96	30.5	22.9	0.68
2513C	3.36	8.3	4.8	4.4	3.6	0.93	8.8	5.3	0.85
3231	3.6	14.9	14.8	15.8	9.1	0.89	20.8	20.8	0.83
3246A	2.8	19.7	18.3	19.6	19.0	0.72	24.7	20.2	0.51
3246B	2.8	37.9	20.2	17.4	17.1	0.84	49.1	34.2	0.43
5185	3.3	23.2	4.0	2.7	2.7	1.00	1.2	1.2	0.99
5495	3.5	35.7	34.4	9.6	9.6	0.95	67.7	60.0	0.33
5584	3.9	30.4	21.5	33.4	17.0	0.88	57.0	39.5	0.52
5764	3.5	32.3	31.8	36.1	32.2	0.89	30.7	27.1	0.66
5778	3.275	14.8	5.8	6.3	6.3	0.90	7.2	7.1	0.89
5925	3.64	3.3	3.3	3.6	3.1	0.96	1.0	0.7	0.99
6219	4.8	23.2	21.7	25.6	22.4	0.86	25.5	24.1	0.76
6272	2.6	14.9	12.3	17.6	12.6	0.96	19.7	17.9	0.78
6374	2.9	2.5	2.1	1.7	1.7	0.99	8.1	7.3	0.85
6478	2.9	40.9	23.3	2.6	1.9	0.98	42.0	30.7	0.49
6551	3.8	10.7	5.1	4.4	4.4	0.93	12.3	11.1	0.87
6555	2.9	8.8	3.0	2.4	1.7	0.97	30.4	27.7	0.48
8011	3.7	13.4	10.4	11.3	10.8	0.87	45.7	24.6	0.20
8015	3.1	2.1	2.1	2.2	1.7	0.99	64.3	59.1	0.35
8116	3.8	50.6	37.9	49.8	35.8	0.76	10.7	10.3	0.88
2364	4.3	38.7	38.7	34.7	34.4	0.67	-	-	-
2850	4.3	23.4	23.2	23.5	23.5	0.79	14.7	13.4	0.84
2867	4.3	15.9	9.8	9.3	8.9	0.90	10.6	10.2	0.83
3063	4.2	28.6	27.4	34.0	29.6	0.72	-	-	-

3073	4.1	41.1	39.8	40.4	39.3	0.88	14.9	12.4	0.82
3074	4.1	36.8	15.3	35.6	15.2	0.86	15.7	14.8	0.85
5155	4.2	31.8	29.5	41.5	25.6	0.83	96.6	96.6	0.23
5376	4.1	24.5	24.3	25.7	24.9	0.83	18.7	15.0	0.75

a) The RMSD of the top 1 and the best among top 10 models from the main-chain models of MAINMAST as well as results after the PULCHRA-MDFF refinement were shown. The coverage value is for the top 1 model after the refinement. Coverage is defined as the fraction of residues in the native structure that are closer than 3.0 Å to the model when superimposed.

b) Results of Rosetta with a 0.8 consensus setting are shown, because this setting gave overall better results than the default setting as shown in Figure S2.

Supplementary Table 4. Modeling summary on the dataset of 43 missing fragments in comparison with RosettaES and RosettaCM

Model ID[a]	Residue Range	Total Residues	RosettaES (Å)[b]	RosettaCM (Å)	MAINMAST (Å)[c]
BPP1	1-111	111	2.5	48.6	3.2
BPP1	119-207	89	3.4	30.8	3.6
BPP1	207-259	53	0.9	4.2	2.1
BPP1	256-283	28	0.7	0.7	2.2
BPP1	283-327	45	2.0	30.5	6.0
FrhA	1-22	22	0.5	6.4	1.4
FrhA	136-165	30	2.1	3.2	0.9
FrhA	187-265	79	1.9	11.6	1.9
FrhA	24-29	26	0.6	0.9	0.6
FrhA	298-339	42	0.8	10.6	2.1
FrhA	337-358	22	0.5	0.5	1.9
FrhB	1-18	18	0.7	1.4	0.7
FrhB	110-143	34	0.7	3.7	1.1
FrhB	179-228	50	0.7	4.1	1.1
FrhB	36-66	31	1.0	1.5	1.4
FrhB	61-87	27	0.8	2.0	2.7
FrhB	87-108	22	1.0	0.8	0.7
FrhG	1-71	71	3.3	28.6	3.5
FrhG	144-172	29	0.9	1.7	1.7
FrhG	192-228	37	3.0	1.6	2.7
FrhG	73-133	61	6.6	5.9	1.6
STIV	1-163	163	16.9	45.9	17.3
STIV	161-252	92	2.3	15.9	3.2
STIV	252-319	68	1.8	11.2	1.3
T20S	13-50	38	1.6	14.6	2.1
T20S	159-220	63	1.6	37.3	4.1
T20S	43-78	36	4.3	2.1	2.2

T20S	88-166	79	1.1	5.9	6.0
TMV	1-11	11	0.7	0.6	0.7
TMV	44-78	35	0.8	0.8	1.1
TMV	78-106	29	3.2	1.5	1.0
TRPV1	1-45	45	6.1	3.0	2.6
TRPV1	111-134	24	1.6	5.6	1.8
TRPV1	128-183	56	4.0	4.8	1.6
TRPV1	205-226	22	3.4	0.7	1.5
TRPV1	226-310	85	7.0	3.5	3.0
TRPV1	66-110	45	1.4	1.7	2.9
VP6	1-81	81	2.6	43.3	3.9
VP6	115-245	131	4.2	38.5	11.0
VP6	243-269	27	2.9	2.0	1.8
VP6	266-299	34	0.7	8.5	1.1
VP6	300-350	51	0.6	3.6	2.3
VP6	349-372	24	1.1	1.0	1.0
VP6	87-118	32	0.6	1.6	1.4
**Average**	-	50.0	2.40	10.30	2.68

a. The dataset is taken from the RosettaES paper (Frenz B et al., Nature Methods, 14: 797-800, 2017), Supplementary Table 1. These proteins have their EM maps of a 3-5 Å resolution and deposited structure models available in EMDB.
b. The results of RosettaES and RosettaCM are taken from the columns of the Best scoring results in the Supplementary Table 1 of the RosettaES paper.
c. Top 5 scoring models from the xMDFF refinement (Greevy R. et al., Acta Crystallogr. D Biol. Crystallogr., 70(Pt 9): 2344-2355, 2017) was averaged and refined using Phenix (Adams et al., Acta Cryst. D66: 213-221, 2010) with a weight between data and restraints set to 100.

Supplementary Figure 1. Comparison with MAINMAST and Pathwalking models for the 40 simulated maps.

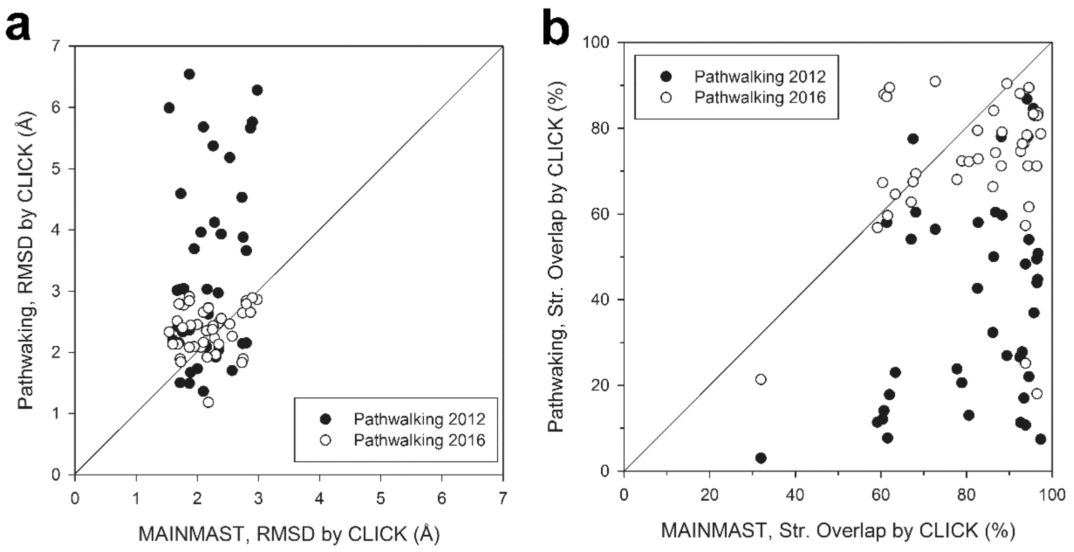

Modeling results of the 40 simulated maps by MAINMAST in comparison with Pathwalking of 2012 and 2016. **a**, local RMSD and **b**, structure overlap of the models by MAINMAST compared with Pathwalking models computed with the CLICK server. For the Pathwalking algorithm, data are taken from the two publications, in 2012 and 2016. For the MAINMAST results, the model with the best threading score among the generated 2688 models were used. Structure overlap by CLICK in the panel **b** is defined as the percentage of residues in a structure placed within 3.5 Å to residues in residues to the other superimposed structure.

Supplementary Figure 2. Top 1 Rosetta models for the 40 simulated maps.

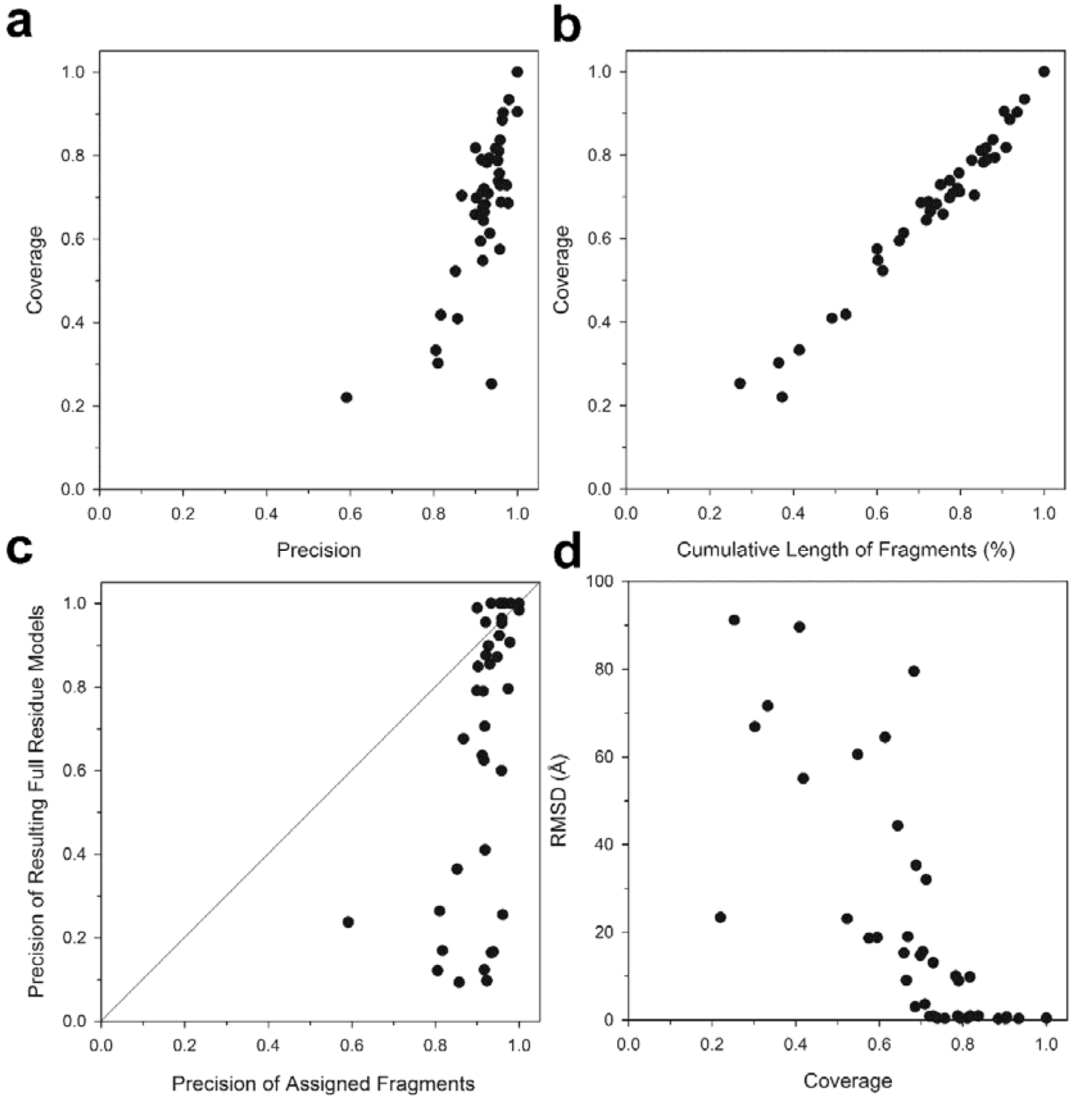

a, coverage (the fraction of residues in the native structure that have some residues in the corresponding model within 2.0 Å) of the models relative to the precision, which is defined as the fraction of residues in a model that are placed within 2.0 Å to some residues in the native structure; **b**, the model coverage plotted against the percentage of the total length of fragments assigned in a map among the full protein length. The observed strong correlation indicates that most of the assigned fragments are accurate but regions modelled to fill gaps between the fragments tend not to be accurate. **c**, Precision (within 2.0 Å) of full residue models relative to the precision (within 2.0 Å) of assigned fragments. The diagonal line is y=x. It is shown here that most of the fragments are assigned with relatively high accuracy, over 0.8, however, the subsequent full-residue modeling step could not make good models, failing to fill gaps precisely. It was also observed that the subsequent modeling step moved some precisely assigned fragments to a wrong place while building a full residue model. **d**, Cα RMSD (Å) of the full residue model plotted relative to the model coverage.

Supplementary Figure 3. Cα RMSD (Å) of models by Rosetta for the 30 real maps.

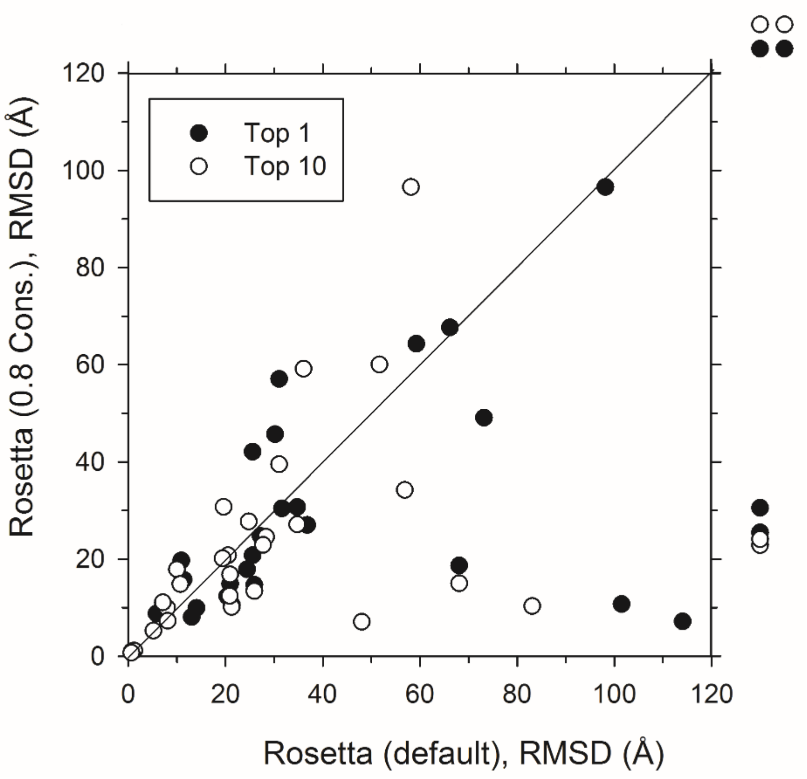

Modeling results for the 30 maps using Rosetta in its default setting (x-axis) and Rosetta (0.8 consensus), where in the Monte Carlo fragment assembly, structures of local regions are kept if 80% of the assigned fragments are placed at the same position. The best RMSD model among all produced (filled circles) and the best RMSD model among top 10 scoring models (empty circles) are shown. Two empty and two filled circles on the right upper corner indicate that there were two structures that were not modelled (no output structures) by both default (filled circles) and with the 0.8 consensus (empty circles) Rosetta. Two more circles filled and empty each indicate that Rosetta (default) did not produce models but Rosetta with 0.8 consensus produced models with an RMSD of about 30 Å for the targets.

In the main text we showed the results by Rosetta with 0.8 consensus because overall this setting produced better (lower RMSD models) for more cases, which are represented by points below the diagonal line.

Supplementary Figure 4. Average accuracy of residue positions relative to the degree of consensus for the models of EMD-2513 and EMD-6272.

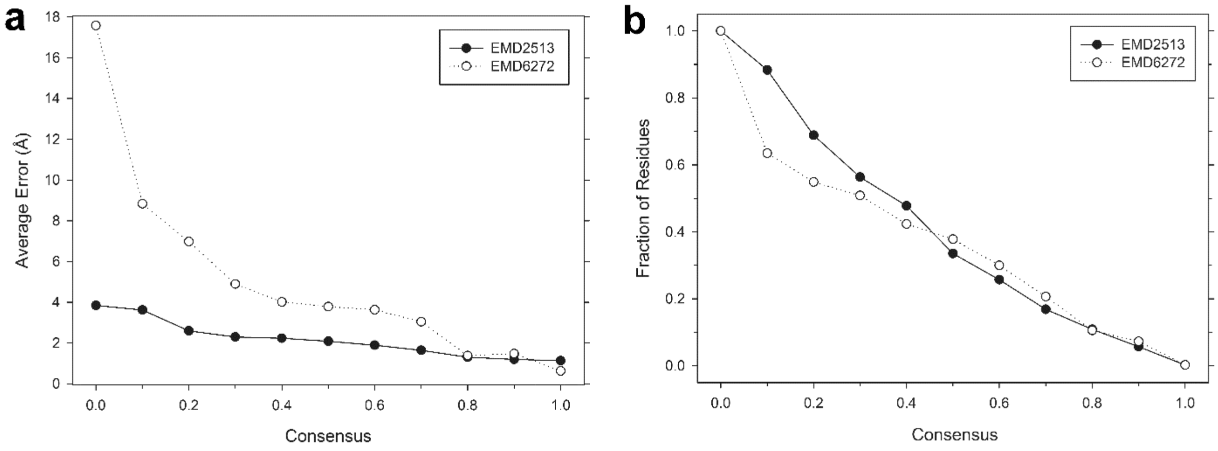

The structures of these two models are shown in Figure 4e and 4f. **a**, error of the Cα positions relative to the degree of consensus among top 100 scoring models. Consensus on the x-axis shows the fraction of the models that have a residue within 3.5 Å. **b**, the fraction of residues in the models that have particular consensus degree among top 100 scoring models.

Supplementary Note 1. Running Rosetta

Rosetta ver. 3.6 (rosetta_bin_linux_2016.31.58825_bundle) was used. We followed the tutorial released on http://dimaiolab.ipd.uw.edu/software/. Almost all the parameters used were as described in the tutorial, but some specific parameters were taken from the following paper:

Wang, R. Y. R., Kudryashev, M., Li, X., Egelman, E. H., Basler, M., Cheng, Y., Baker, D., & DiMaio, F. (2015). De novo protein structure determination from near-atomic-resolution cryo-EM maps. *Nature Methods*, 12(4), 335-338.

First, fragment structures for the query protein were generated on the Robetta website:

http://robetta.bakerlab.org/fragmentqueue.jsp

1. Local Fragment search in an input EM map using denovo_density.

This procedure searches the density map for each sequence-predicted backbone fragment generated in the previous step.

```
$ROSETTA3/source/bin/denovo_density.linuxgccrelease \
    -in::file::fasta ./target.seq \
    -fragfile ./aat000_09_05.200_v1_3.txt \
    -startmodel ./start_model.pdb \
    -mapfile ./MAP.mrc \
    -num_frags 25 \
    -n_to_search 2000 -n_filtered 2500 -n_output 50 \
    -bw 16 \
    -atom_mask_min 2 \
    -atom_mask 3 \
    -clust_radius 2 \
    -clust_oversample 4 \
    -movestep 2 \
    -delR 2 \
    -frag_dens 0.8 \
    -ncyc 3 \
    -min_bb false \
    -pos $1 \
    -out:file:silent round$2/fragment.$1.silent
```

The parameter “-n_to_search 2000 -n_filtered 2500 -n_output 50” was used following the paper by Wang et al. mentioned above.

2. Placed fragment scoring using denovo_density:

This step score the placement of fragments for compatibility to the EM map.

```
$ROSETTA3/source/bin/denovo_density.linuxgccrelease \
    -mode score \
    -in::file::silent round1/fragment*silent \
    -scorefile round1/scores1 \
    -n_matches 50
```

3. Monte Carlo fragment assembly using denovo_density:

This step generates “maximally consistent” fragment assembly in the map.

```
$ROSETTA3/source/bin/denovo_density.linuxgccrelease \
    -mode assemble \
    -nstruct 40 \
    -in::file::silent round1/fragment*silent \
    -scorefile round1/scores1 \
    -assembly_weights 4 20 6 \
    -null_weight -150 \
    -out:file:silent round1/assembled.$1 \
    -scale_cycles 1 \
    -mute core
```

Following the paper, we performed total 2,000 trajectories (i.e running the above script 50 times in parallel, 40 struct * 50 times = 2000 trajectories) for each round.

4. Consensus assignment using denovo_density:

This step is to identify the consensus assignment from the lower-scoring Monte Carlo trajectories.

```
$ROSETTA3/source/bin/denovo_density.linuxgccrelease \
    -mode consensus \
    -in::file::silent round1/assembled.*silent \
    -consensus_frac 1.0 -energy_cut 0.05 \
    -mute core
```

In our manuscript, we also used a 0.8 consensus setting by “-consensus_frac 0.8” because 0.8 gave better results for many cases.

If the assigned backbone residues are less than 70% of the target protein or the coverage is not converged, we iterate the four (1-4) steps.

5. Running RosettaCM

Following the paper "Wang, R. Y. R., Kudryashev, M., Li, X., Egelman, E. H., Basler, M., Cheng, Y., Baker, D., & DiMaio, F. (2015)", this step is applied to fill gaps where the fragments were not assigned by denovo_density to complete a model and to refine the overall model structure.

```
$ROSETTA3/source/bin/rosetta_scripts.linuxgccrelease \
 -database $ROSETTA3/database/ \
 -in:file:fasta target.seq \
 -parser:protocol rosettaCM.xml \
 -nstruct 50 \
 -relax:minimize_bond_angles \
 -relax:min_type lbfgs_armijo_nonmonotone \
 -relax:jump_move true \
 -relax:default_repeats 3 \
 -relax:dualspace \
 -out::suffix _$1 \
 -edensity::mapfile MAP.mrc \
 -edensity::mapreso 5.0 \
 -edensity::cryoem_scatterers \
 -default_max_cycles 200
```

Total 1,000 full-atom models are generated by RosettaCM.
